# Current updates on arrhythmia within Timothy syndrome: genetics, mechanisms and therapeutics

**DOI:** 10.1017/erm.2023.11

**Published:** 2023-05-03

**Authors:** Congshan Jiang, Yanmin Zhang

**Affiliations:** 1 National Regional Children's Medical Centre (Northwest), Key Laboratory of Precision Medicine to Pediatric Diseases of Shaanxi Province, Xi'an Key Laboratory of Children's Health and Diseases, Shaanxi Institute for Pediatric Diseases, Xi'an Children's Hospital, Affiliated Children's Hospital of Xi'an Jiaotong University, Xi'an, Shaanxi 710003, China; 2 Department of Cardiology, Xi'an Children's Hospital, Affiliated Children's Hospital of Xi'an Jiaotong University, Xi'an, Shaanxi 710003, China

**Keywords:** Arrhythmia, *CACNA1C*, clinical therapeutics, LTCC, pathogenesis, prolonged corrected QT interval, Timothy syndrome

## Abstract

Timothy syndrome (TS), characterised by multiple system malfunction especially the prolonged corrected QT interval and synchronised appearance of hand/foot syndactyly, is an extremely rare disease affecting early life with devastating arrhythmia. In this work, firstly, the various mutations in causative gene *CACNA1C* encoding cardiac L-type voltage-gated calcium channel (LTCC), regard with the genetic pathogeny and nomenclature of TS are reviewed. Secondly, the expression profile and function of *CACNA1C* gene encoding Ca_v_1.2 proteins, and its gain-of-function mutation in TS leading to multiple organ disease phenotypes especially arrhythmia are discussed. More importantly, we focus on the altered molecular mechanism underlying arrhythmia in TS, and discuss about how LTCC malfunction in TS can cause disorganised calcium handling with excessive intracellular calcium and its triggered dysregulated excitation–transcription coupling. In addition, current therapeutics for TS cardiac phenotypes including LTCC blockers, beta-adrenergic blocking agents, sodium channel blocker, multichannel inhibitors and pacemakers are summarised. Eventually, the research strategy using patient-specific induced pluripotent stem cells is recommended as one of the promising future directions for developing therapeutic approaches. This review updates our understanding on the research progress and future avenues to study the genetics and molecular mechanism underlying the pathogenesis of devastating arrhythmia within TS, and provides novel insights for developing therapeutic measures.

## Introduction

Timothy syndrome (TS), also referred to as long QT syndrome type 8 (LQT8), was widely recognised as an extremely rare disease following the Mendelian rules of autosomal-dominant inheritance (Online Mendelian Inheritance in Man database, OMIM No. 601005). The disease prevalence was registered as less than 1 in 1 million according to the archive in the Orphanet database (https://www.orpha.net) (Ref. 1). More importantly, TS exhibited very early onset ages for developing neonates. Prolonged corrected QT interval (QTc) and the synchronised appearance of hand/foot syndactyly were well accepted as the signature clinical characteristics of TS patients since this disorder was primarily described in 1992 (Ref. 2). To date, much more attention have been paid, and the understanding of TS was further refreshed as a multisystem disorder with various extra-cardiac disease phenotypes such as dysmorphic craniofacial features, neurodevelopmental malfunction, immune dysregulation and so on (Ref. 3). With arrhythmia, ventricular fibrillation and highly increased risk for sudden cardiac death (SCD), TS patients diagnosed in their early ages are hardly expected to survive to adulthood (Ref. 4). During the study of Splawski *et al*. (Ref. 5), 59% of the TS children were facing the life expectancy of <2.5 years. Ventricular tachyarrhythmia was claimed to confer the leading risk of death in TS patients (Ref. 6).

Here, we updated our understanding on the research progress and future avenues to study the genetic and molecular pathogenesis of devastating arrhythmia in TS, and aimed to provide novel insights for the development of therapeutic measures.

## Genetic pathogeny and nomenclature of TS

### Genetic pathogeny of TS

According to early findings of Reichenbach *et al*. in 1992 (Ref. 2) and Marks *et al*. in 1995 (Ref. 6), the clinical manifestation of a novel ‘heart-hand’ syndrome with LQT and syndactyly were gradually noted by the physicians. Although the phenotype diagnosis of heart-hand abnormality was recognised long time ago, the genetic aetiology and pathogenesis have troubled the clinicians. Later in 2004, the official name of ‘Timothy syndrome’ was introduced by Splawski *et al*. (Ref. 7), characterised by the combination of QTc longer than 480 ms and one or more clinical phenotypes including (1) syndactyly; (2) TS-related facial and dental abnormality; (3) neurodevelopment disorders such as autism spectrum disorder or congenital heart disease (CHD). With the rapid development of genotyping, the rare genetic variant of TS patients was preliminarily uncovered then, which helped us start understanding the origin of so many unfavourable clinical consequences. It was found that the mutation within *CACNA1C* gene encoding the Ca_v_1.2 (the crucial pore-forming transmembrane) subunit of the cardiac L-type voltage-gated calcium channel (LTCC) is mainly responsible for TS onset. The most classical mutations include the *de novo* substitution causing missense p.Gly406Arg (short as G406R) within the exon 8 (Ref. 5) or 8a (alternative splicing isoform) (Ref. 7) of *CACNA1C* transcript. Other *CACNA1C* gene variants are also increasingly discovered as highly implicated in the potential pathogenesis of arrhythmia-related disorders including TS, with the help of advanced techniques especially whole-exome sequencing (WES).

### Conventional nomenclature of TS

TS was previously designated into various types. Among them, type 1 TS (TS1) patients with the p.G406R mutation in exon 8A displayed signature syndactyly (Ref. 7). Although the type 2 TS (TS2) patients with other mutations such as p.G406R and p.Gly402Ser (short as G402S) in exon 8 were found spared for the syndactyly phenotype, they may display more severe cardiac malfunction (Refs 5, 8, 9). Interestingly, the splicing combination choosing either the exon 8 or exon 8A was found mutually exclusive within the mRNA transcripts. These exons were found both widely expressed in multiple tissues including heart and brain. According to Northern blotting results of Splawski *et al*. (Refs 5, 7), the expression ratio varies, for example, although both exons were highly expressed in heart, the expression of exon 8A splice variant seemed more abundant in aorta, while less in brain, compared with exon 8. Although these two exons with the same length (104 bp) but one-third variation for the coded amino acids (aa), share the very crucial common code for Gly at the 406th aa, which were both reported as important mutations (into Arg) in both the TS1 and TS2 (Ref. 10). As for the crucial cardiac manifestation, penetrance of QTc interval prolongation in TS G406R mutation carriers was found as high as 100% in some studies (Refs 5, 7, 11). Besides, there are also sporadic cases of ‘cardiac-only Timothy syndrome (COTS)’ reported carrying other non-classical TS variants such as p.Arg518Cys (shorted as R518C, in exon 12) (Ref. 12). Although TS patients exhibit complicated multi-system disorders, prolonged QTc interval on electrocardiogram (ECG) characterised arrhythmia is basically considered as mostly the obligatory disease phenotype of TS except for some cases such as carrying the atypical *CACNA1C* p.Arg1024Gly (shorted as R1024G) mutation (Ref. 13). Rodan *et al*. (Ref. 14) also reported some patients with primarily neurological phenotypes carrying atypical mutations including *CACNA1C* L614P, L614R, L657F and L1408V, contributing to loss-of-function (LOF), gain-of-function (GOF) and even neutral effect on channel function. Moreover, with the potent techniques including WES and systems biology, many other rarely reported variants within *CACNA1C* gene were discovered implicated in cardiac malfunction (Refs 9, 15). Generally speaking, as far as we know, TS is still divided as TS1, TS2 or the so-called atypical TS, which carries other *CACNA1C* mutations except for the typical mutations in TS1 and TS2 with multi-organ healthy issues (Ref. 1). Although in some of these *CACNA1C* variants, TS-related extra-cardiac phenotype was not always found, only with QT prolongation and arrhythmia, thus described as non-TS LQT8 (Ref. 16).

### Latest update on the genetic pathology within TS

For the classification, researchers keep updating and are starting to discuss on the relative open-minded opinion about whether the TS3 and so on could be reserved for the expanding TS causative gene variants which are yet to be uncovered and still controversial (Ref. 10). In the commonly applied Human Gene Mutation Database (HGMD) (Ref. 17) (accessed on 5 December 2022), the documented gene mutations contributing to the disease/phenotype term of ‘Timothy Syndrome’ (TS1) are increasing, containing dozens of missense mutations such as p.P381S, G402S, G406R, E407A, R412M, G419R, S643F, C1021R, C1021Y, I1166T, A1473G, M1476R and V1518E in NP_000710.5 (translated by the NM_000719.7 transcript). Although only two gene mutations are archived as contributing to the disease/phenotype term of ‘Timothy Syndrome 2’ (TS2), including p.G402S and G406R in NP_001161097.1 (translated by the NM_001167625.2 transcript), there are also a couple of other genetic mutations considered as contributing to the ‘Timothy-like syndrome’.

The prevalence of TS is difficult to calculate, while such efforts were still paid in sporadic studies, for example, it was reported as 0.19 TS1 per 10,000 newborns in a single hospital from Mexico during 2009–2013 (Ref. 18). Possibly because of the extreme low morbidity, until 5th January in 2023, when searching with the key word ‘Timothy syndrome’, the publications archived in PubMed database are as few as 172 items, which is quite a small quantity. Although TS has always been a major burden troubling both the clinicians and the entire family of paediatric patients, its highly limited clinical resources, make TS really difficult to study.

## Molecular mechanisms underlying the *CACNA1C* gene dysfunction for arrhythmia within TS

### Expression profile and function of Ca_v_1.2 (*CACNA1C*)

**Figure 1.**
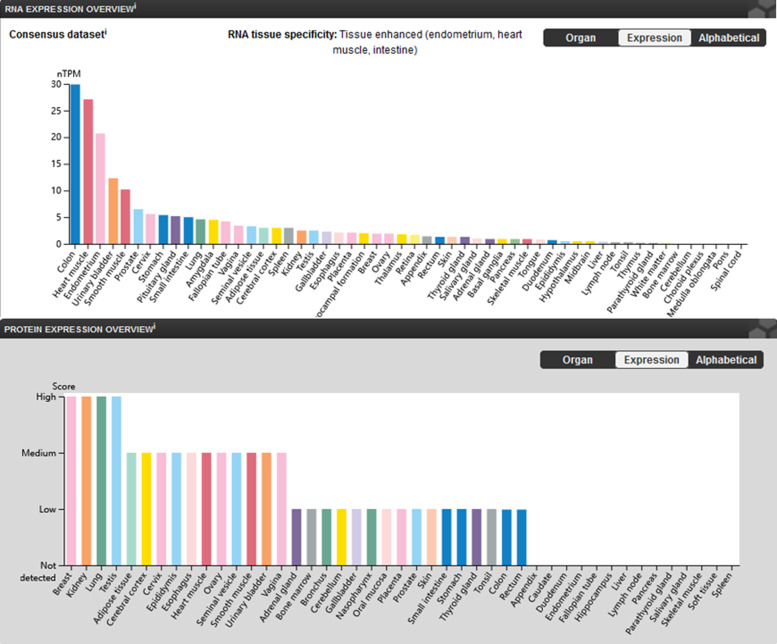
Tissue-specific protein expression of Ca_v_1.2. Image available from the Human Protein Atlas (v21.1.proteinatlas.org, https://www.proteinatlas.org/ENSG00000151067-CACNA1C/tissue). The consensus dataset consists of normalised mRNA expression (nTPM) levels for 55 tissue types, created by combining the HPA and GTEx transcriptomics datasets. Protein expression data are shown for each of the 44 tissues.

Because of the important role of calcium in excitatory tissues especially the heart and brain, it is easily understood that the malfunction of *CACNA1C* gene in heterozygous individual is causative and enough for the devastating cardiological consequence especially the arrhythmic disorders, as well as neurological disorders. However, as we know, disease phenotypes of TS are much more. According to the data from Human Protein Atlas (Ref. 19), *CACNA1C* (coding gene for Ca_v_1.2) was widely expressed in multiple tissues (Fig. 1). The mRNA expression profiling dataset showed that *CACNA1C* mRNA is the second highest expressed in human heart muscle among 55 tissues. Its protein expression is also the top one-third ranking among 44 tissues. Its genetic mutation could thus result in the disease phenotype within multiple organs which are responsible for the clinical constellation of TS.

*CACNA1C*-encoding *α*_1_-subunit of Ca_v_1.2, which is the major component of the LTCC functional channel complex, is crucial for calcium ion transportation in various cells including cardiomyocytes (CMs) (Ref. 20). In Ca_v_1.2 channel, there are four repeats (nos. I–IV) of six transmembrane segments (named S1–S6) (Ref. 21). The *α*-subunit interaction domain (AID) binding area locates at the end of IS6 (first of the four domains, no. 6 transmembrane segment) region, upstream of I–II domain intracellular region. AID regulates the voltage-dependent activation and inactivation of channel (Ref. 22).

### GOF mutation of *CACNA1C* gene encoding Ca_v_1.2 proteins in TS leads to multiple organ disease phenotype

In TS1 and TS2, G406R mutation in exon 8a or 8 are both located between IS6 and AID, thus is crucial to maintain normal channel inactivation (Refs 5, 7). Splawski *et al*. (Ref. 7) achieved the first breakthrough in 2004 to propose that the *CACNA1C* GOF mutation could bring up prolonged calcium current and thus delayed repolarisation in CMs, and such defected voltage-dependent inactivation (VDI) of Ca_v_1.2. In CMs, persistent Ca^2+^ influx and intracellular Ca^2+^ overload results in prolongation of action potential duration, early after depolarization (EAD) and eventually life-threatening cardiac disorders. The arrhythmia phenotype of TS is thus resultant, including longer QTc, 2:1 atrioventricular (AV) block, T-wave alternans (TWA), CHD, myocardial hypertrophy and so on (Refs 5, 7). The prolongation of QT interval was also reported to cause the functional 2:1 AV block (Refs 5, 7, 23). Moreover, the TWA trait was found to be associated with the QTc prolongation within syndactyly patients in Chinese children (Ref. 24).

Besides the important pathogenic mechanism in TS, previous studies also found that functional gain and loss mutations of *CACNA1C* gene encoding Ca_v_1.2 proteins are often associated with hereditary arrhythmia syndromes including non-TS long QT syndrome, Brugada syndrome and so on (Ref. 25), which demonstrated that the strong implication of dysregulation of *CACNA1C* gene function in the arrhythmia phenotype. On the contrary, mutations with the LOF of Ca_v_1.2 channel were found to exhibit shorter QT intervals (Ref. 26). Such evidence further demonstrated the important implication of *CACNA1C* in arrhythmia phenotype. This could be the very reason why the majority of the previous TS-related studies focused on the relevance of TS with arrhythmia.

It was well accepted that the *CACNA1C* GOF mutation is capable to cause major disorders in electrically excitable tissues. In neurons, Ca^2+^ influx increases resulting in dysregulation of nuclear gene expression and neuropsychiatric phenotype. In vitro studies showed that TS-related *CACNA1C* GOF could bring out damaged radial neuron migration in developing mouse neurons, which might contribute to a consequence of cortical malformation in the developing brain (Ref. 27). In *Caenorhabditis elegans*, its mutation was found to be related to defected axon termination and behaviour (Ref. 28). These cellular malfunctions eventually contribute to the collective neurodevelopmental disorders.

Besides, the excitability and exocytosis in chromaffin cells with TS2 mutation was impaired because of the increased window calcium current in mice (Ref. 29). However, the intervention of the calcium-dependent insulin secretion of islet beta cells could be the mechanism of intermittent hypoglycaemia found in some of the TS patients (Ref. 7).

In non-excitatory cells and tissues, the role of *CACNA1C* GOF was also emerging. During the development of zebrafish *CACNA1C* was found implicated in the process of hypertrophy and hyperplasia in the cell subtypes within mandible during jaw development, which might help explain the craniofacial abnormalities displayed in TS patients (Ref. 30). *CACNA1C* G406R mice displayed delayed anagen and impaired follicle tissue generation, leading to baldness (Ref. 31). By affecting the Ca^2+^-dependent maturation and differentiation of T lymphocytes, it leads to immune dysfunction, causing recurrent infection usually seen in TS patients (Ref. 7).

**Figure 2.**
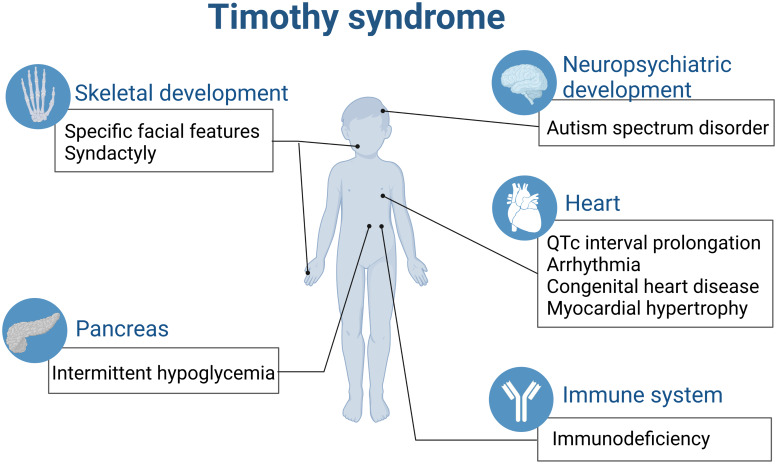
TS is a paediatric rare disease involving multiple organs (created with biorender.com). QTc, corrected QT interval.

All the above-mentioned effects contribute to the complicated multiple system malfunction caused by single gene of *CACNA1C* in TS (Fig. 2).

### Molecular mechanism underlying arrhythmia in TS

For the TS arrhythmia mechanism study, there have been a few convincing evidence from induced pluripotent stem cell (iPSC)-CM studies. We are also glad that mounting evidence has been accumulated for the study of extra-cardiac phenotype in TS. Besides, many interesting molecular pathway findings published in neuroscience journals are actually carried out in the engineering cell models such as HEK293 cells. Hence these precious pathway findings are also included and further discussed in this review about the pro-cardiomyopathic and proarrhythmic role of *CACNA1C* GOF in TS pathogenesis.

Firstly, LTCC malfunction in TS causes disorganised calcium handling with excessive intracellular Ca^2+^ and subsequent malfunction. *CACNA1C* plays the vital role in CMs, and disorganised calcium handling could cause excessive intracellular Ca^2+^. Besides, calcium dysregulation could trigger mitochondrial energy malfunction. TS-related disorganised calcium handling exhibited as excessive calcium influx and sarcoplasmic reticulum Ca^2+^ cycling, besides calmodulin-dependent protein kinase II (CaMKII) was found necessary for the proarrhythmic consequences of compromised VDI for Ca_v_1.2 (G406R in exon 8), as well as increased peak *I*_Ca_ in primary rat ventricular myocytes (Ref. 32).

Besides the direct impact on the electrical properties of the membrane, the excessive intracellular calcium also triggers dysregulated excitation–transcription (E–T) coupling. As we mentioned above, extensive attention has been always paid to the role of *CACNA1C* in important intracellular signalling cascades including the excitation–contraction (E–C) coupling for cardiomyocyte contraction, as well as the excitation–secretion (E-S) coupling in endocrine system. Although *CACNA1C* also plays an important role in the regulation of transcription and phosphorylation, known as the E–T coupling. Hence, the abnormal function of pleiotropic *CACNA1C* mainly could mediate the pro-cardiomyopathic and proarrhythmic events in TS probably by dysregulating E–C and E–T coupling (Ref. 33).

Ca_v_1.2 binds with protein kinase A, calcium/calmodulin (CaM)/CaMKII and other proteins for signalling regulation. It is found that Ca_v_1.2 activates inward calcium current to participate in transcriptional regulation (Ref. 11). Ca_v_1.2 with *CACNA1C* G406R mutation in HEK293 cells was found as GOF with spontaneous protein expression of cFOS, MeCP2 and phosphorylated CREB (Ref. 34). The cFOS, as an activator protein 1 component, is the immediate early gene, participating in the positive regulation at the transcriptional level on various genes in CMs (Refs 35, 36). MeCP2 and activated CREB are also previously known as strongly implicated in cardiac development and dysfunction (Refs 37, 38). The regulation on even single one of these key transcriptional factors could trigger cascades of downstream signalling target molecules in cardiac tissues, thus the capacity to affect on all of them is overwhelming to participate in the pathogenesis of devastating arrhythmia in TS.

**Figure 3.**
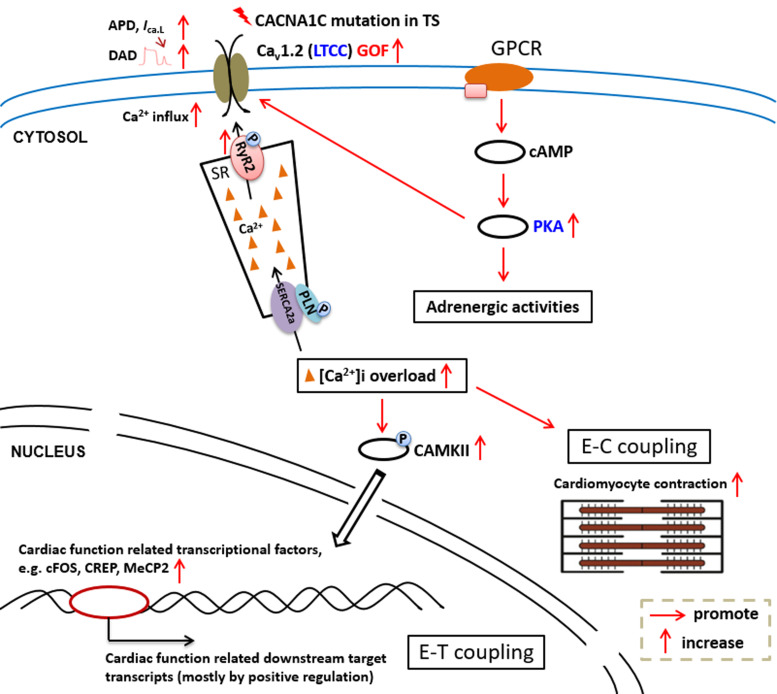
Current understanding of the dysregulated molecular signalling underlying pro-cardiomyopathic and proarrhythmic pathogenesis in TS. APD, action potential duration; DAD, delayed after depolarisations; E–C, excitation–contraction; E–T, excitation–transcription; GOF, gain-of-function; GPCR, G-protein-coupled receptor; LTCC, L-type voltage-gated calcium channel; PKA, protein kinase A; RyR2, type 2 ryanodine receptor; SR, sarcoplasmic reticulum; PLN, phospholamban.

These above-mentioned molecular signallings play vital and interactive roles in the pathogenesis of TS (summarised in Fig. 3), and provide important understanding for the action of *CACNA1C*.

## Current therapeutics for cardiac phenotypes within TS

Since TS holds the major risk of life-threatening distinguishing cardiac phenotypes including markedly prolonged QTc, bradycardia, 2:1 AV block, delayed diagnosis is relatively rare during clinical practice nowadays. Although the genetic and functional origin of TS is presently better understood, there are still huge challenges in clinical practice for the optimised therapy. Or we can even say much of this part is elusive. Currently, TS treatment is mainly aimed at cardiac phenotype, including drugs, such as beta-receptor blocker, sodium channel blocker mexiletine, calcium channel blocker, but the clinical efficacy is limited (Ref. 4). Especially, given both the LQT and extra-cardiac multi-organ phenotypes, generally speaking, the prognosis of TS after current clinical treatment is considered very disappointing.

### LTCC blockers fail our expectation

According to the function of *CACNA1C* gene as we discussed above, the initial strategy for pharmotherapeutical treatment was naturally designed to block the LTCC in TS. However, during the clinical studies, the LTCC blockers were found to be unsatisfactory. Verapamil plays a pharmacological role as a Ca_v_1.2 channel blocker (*I*_Ca_ antagonist) and was reported to well control one case of TS cardiac tachycardia, although not capable to significantly decrease the QTc interval (Ref. 39). Shah *et al*. also reported that verapamil could not always bring the ventricular arrhythmias under control, unless with the help of ranolazine (multi-target ion-channel blocker) (Ref. 40). Moreover, verapamil sometimes even worsened the TdP in TS2 infants (Ref. 41). Other LTCC blockers, such as diltiazem, increases TWA and 2:1 AV block (Ref. 40). Such findings are very confounding. It was hypothesised that the mutation might change the structure and energy of the ion channel, thus altering the state-dependent block by dihydropyridines, verapamil, etc. (Refs 7, 42, 43).

### Beta-adrenergic blocking agents are favourable in TS therapy

Nowadays, beta-adrenergic blocking agents (also named beta-blockers) have been applied since as early as 1995 (Ref. 6), and they are still the mainstay during TS therapy. For example, the propranolol-applied TS1 infant was reported without arrhythmias any longer while side effects such as hypoglycaemia should be taken care of (Ref. 44). There was one case report in which the clinical condition of a TS1 baby girl was followed up since her foetal echocardiography was found abnormal until the age of 2. Her poor cardiac condition brought her to the medication plan of mexiletine administration on the very first postnatal day (PD) of her life aided with epicardial VVI-pacemaker on PD4. At 5 months old (4.5 kg of body weight), her VVI-pacemaker was surgically replaced with the extra-cardiac cardioverter-defibrillator. At 21 months old, the patient was added with a propranolol prescription. And during the follow-up, this patient was no longer troubled with life-threating arrhythmia, and both the mexiletine and propranolol was not discovered with safety concern applied to this paediatric patient at such young age, although the extra-cardiac disorders were not improved (Ref. 45).

However in some other cases, the prognosis of TS patients, while on beta-blocker medications, is still considered as not favourable (Ref. 46). In some cases, the beta-blockers such as propranolol performed very poor in controlling the prolonged QTc, yet the mechanism is without a clue (Refs 47, 48). The effect of 2:1 AV block from isoproterenol was also considered as not sustained enough (Ref. 48). Besides the abnormal ECG manifestation, SCD attack was also very concerning and life-threatening. However, multicentre studies including Dufendach and others on 17 TS cases showed that sole use of beta-receptor blockers such as nadolol, propranolol and atenolol do not protect SCD in these studies (Refs 4, 49). Moreover, for patients with TS, because of increased calcium-dependent insulin secretion of islets beta cells, beta-blocker treatment is more likely to lead to hypoglycaemia, with an incidence of 72% (Ref. 50).

### Sodium channel blocker and multichannel inhibitors are emerging in TS therapy

The TS therapy using sodium channel blocker turned out with some scattered good results. Gao *et al*. (Ref. 47) reported that mexiletine as a late sodium current (*I*_Na,L_) blocker could improve the cardiac malfunctions displayed in ECG including attenuating the prolonged QTc, eliminating the 2:1 AV block and TWA in a Chinese TS1 girl of 2 years old. A four and half years old Turkey girl with TS (G406R, although in which exon, either the exon 8 or 8a, is not mentioned in this study) was reported to have a satisfactory therapeutic effect on her abnormal QTc interval and TWA following mexiletine treatment (Ref. 51). During a follow-up study from 2011 to 2019, the addition of mexiletine to the routine nadolol (beta-blocker) administration was observed to shorten the QTc and ameliorate the shock frequency in a TS2 patient (Ref. 52). The above-mentioned mexiletine mediation was found to shorten the QTc possibly via its role as class IB sodium channel blocker in a BayK 8644-treated ventricular wedge in vitro *I*_Ca_ GOF model, which aroused our attention on multifunctional inhibitors (Ref. 33). However, the multichannel inhibitors, such as ranolazine affecting both the *I*_Ca_ and *I*_Na,L_, were reported to successfully treat a 13-year-old boy with TS2 carrying the G402S mutation and keep him shock-free (Ref. 53).

### Pacemakers aid the medications in TS therapy

Besides medications, the pacemakers, such as implantable cardioverter-defibrillator (ICD) were also available, and left cardiac sympathetic denervation (LCSD) could also be used. It was reported that the epicardial ICD could be chosen as a supplement for medication (Ref. 54), when the TS patients' condition (weight and size) permits. LCSD was reported to improve the frequency of shock in TS2 patients (Ref. 52). For now, it was accepted that with the combination of such implantable defibrillator device with the original medicational programme could be a default choice for TS patients (Ref. 55).

### Present status of TS therapeutics

In short, although the TS patients were widely considered deem to be at an extreme high risk of arrhythmias together with many other disorders during the entire life, the present single usage of beta-blockers, late sodium channel blockers and/or pacing has contributed a hopeful prognosis to some extent though limited, still buying us some extra time for the pharmaceutical development. Moreover, under such medication programme, the extra-cardiac manifestation such as impaired neurodevelopment was not mended so far. Randomised controlled clinical trials are rather unavailable for TS which is a paediatric rare disease with a tiny patient population. Especially considering the major cases in infants, drug trials are more infeasible. This is why the present clinical management of TS remains unsatisfactory. To speed up the progress, the experimental models for TS study are much in need.

## Translational implications/clinical applications

Addressed as the great difficulty in many important case reports (Ref. 56), although the causal relationship between specific genotype and the clinical phenotype was crucial, it is yet difficult to further evaluate ideal therapy for each specific genetic variant without a suitable experimental model for TS. This is why massive attempts have been made in this direction.

Considering protein homology, human-derived models are always the first choice. The human iPSCs were greatly advocated since they not only provide the in vitro model for TS mimicking, but also offer the possibility to study the early stages of human organ development thus resemble the paediatric and infant condition which is different from that in adults at both the electrophysiological and molecular levels. In 2011, Yazawa captured the irregular contraction, excess Ca^2+^ influx and abnormal calcium transients in ventricular-like iPSC-CMs from TS patients, which is very crucial for the LQT in vitro study (Ref. 57). In 2013, Yazawa *et al*. further established five TS patient-derived iPSC lines, and differentiated them into iPSC-CMs. Their functional evidence supported that the TS-iPSC-CMs could be useful models to characterise cardiac phenotypes in TS (Ref. 58). Moreover, now there have developed a supervised machine learning algorithm based on the iPSC-CMs derived from healthy donors and TS patients (Ref. 59). And it was reported that the model trained on the various parameters such as beat frequency, interbeat variability, peak width, etc. within the contractility traces was able to apply to the unknown data and predict potential proarrhythmic events or drug–target interactions. Besides the classical TS variants, the iPSC-CM studies on other *CACNA1C* mutation were also very popular. In 2019 Chavali *et al*. validated the patient-derived iPSC-CMs with *CACNA1C* N639T mutation serves as an ideal model to determine AP and ion channel for arrhythmia functional assay side by side with its isogenetic control (Ref. 60). In 2019, Estes *et al*. established *CACNA1C* R518C iPSC-CMs to study the AP and LTCC function (Ref. 61).

Cells from TS individuals have defects in calcium (Ca^2+^) signalling and activity-dependent gene expression. Heterozygous expression cell lines, based on iPSCs of patients are a potent platform to study the gating mechanism of channel activation and inactivation, and test the effect of channel blockers. From our perspective, since the iPSCs have the same genetic material as the donor and reproduce the disease phenotype, the most attractive potential may be to use iPSC instead of individuals to predict the treatment response of specific drugs. Moreover, by comparing the patient-specific iPSC toxicity with the standard in vitro toxicity index, clinicians can have scientific basis to predict the treatment risk ratio and determine the best drug for patients (Ref. 62). This characteristic is especially suitable for the study of the pathogenesis and treatment strategies of paediatric rare diseases such as TS.

**Figure 4.**
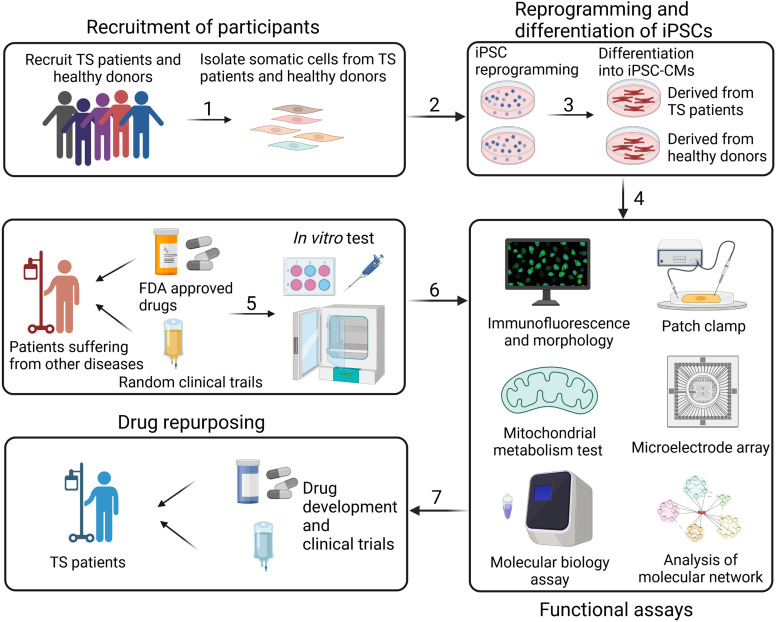
Research strategy of drug development for TS with patient-specific iPSC-CMs (created with biorender.com). CM, cardiomyocyte; FDA, Food and Drug Administration; iPSC, induced pluripotent stem cells; TS, Timothy syndrome.

As generally recognised, randomised controlled clinical trials are rather unavailable for TS which is a paediatric rare disease with a tiny patient population. Given the major cases in infants, clinical trials for potential therapy are more infeasible. Encouraging progresses have been made highly thanks to the promising research strategy using patient-specific iPSCs. iPSCs carrying the patient-specific genetic information, are highly reproductive in vitro, capable to differentiate into various cell types including the iPSC-CMs and even cardiac organoids, which provides a unique research platform (Fig. 4) for screening potential medications, evaluating the mechanism of drug cardiotoxicity, discovering risk populations, predicting drug responses as well as designing novel therapy. iPSC-CM differentiation could be completed within a few months since this mutation is found during clinical practice, the pathogenesis is quickly determined and the feasibility of individualised and accurate treatment is guaranteed (Ref. 60).

However, iPSC-CMs were previously concerned for its immaturity after differentiation, the additional procedures were needed for further maturation to fully mimic the characteristics in morphology, electrophysiological function and molecular expression. Especially the short of classic spike result in the doubt on the capacity of iPSC-CM to study arrhythmia-related phenotypes to recapitulate cardiac arrhythmias for LQT studies (Ref. 63). More efforts are called for the optimising of iPSC-CMs with suitable maturation status.

Moreover, the comprehensive application of advanced techniques in cell culture and differentiation, high-throughput analysing platform, unbiased multi-omics screen, bioinformatics and tissue engineering, brings new prospective for the understanding of TS pathogenesis, ensures the promising potential of iPSCs in the application within the precision and translational medicine of TS.

## Expert and topical summary

In summary, current therapeutics for cardiac phenotypes within TS including LTCC blockers, beta-adrenergic blocking agents, sodium channel blocker, multichannel inhibitors and pacemakers are so far helpful. Meanwhile, the research strategy using patient-specific iPSCs is recommended as crucial future direction for developing more promising therapeutic approaches and better understanding the molecular mechanism underlying the pathogenesis of devastating arrhythmia within TS.
